# Palliative care for HIV in the era of antiretroviral therapy availability: perspectives of nurses in Lesotho

**DOI:** 10.1186/1472-684X-8-11

**Published:** 2009-08-15

**Authors:** Megan E Kell, John D Walley

**Affiliations:** 1 Nuffield Centre of International Health and Development, Leeds Institute of Health Sciences, University of Leeds, Leeds, UK

## Abstract

**Background:**

Southern Africa is disproportionately affected by the HIV/AIDS epidemic. In Lesotho 23% of adults are HIV-positive, and only 26% of those in need are accessing antiretroviral treatment (ART). Consequently, about 18,000 people die from AIDS each year. In this situation, palliative care is needed towards the end of life, but is also recommended throughout the HIV disease trajectory. The World Health Organisation (WHO) has produced the Integrated Management of Adolescent and Adult Illness (IMAI) guidelines, which includes a palliative care guidebook (as well as acute and chronic ART guidebooks). IMAI aims to facilitate the implementation of integrated HIV/AIDS care in resource-poor areas. The opinions of health workers towards this integrated approach to care and the use of IMAI has not been considered in previous research studies. This paper therefore aims to address some of these issues.

**Methods:**

Semi-structured interviews were conducted with six key informants and ten nurses in Lesotho. The interviews were transcribed verbatim and analysed using content thematic analysis.

**Results:**

Many nurses described palliative care as synonymous with chronic care and felt that palliative care is necessary for HIV-positive patients despite the introduction of ART. It was thought that the approach taken should be holistic and integrated throughout the disease trajectory. Pain management was noted to be a particular area of need for palliative care, and it was suggested that this could be improved in Lesotho. The IMAI guidelines were thought to be useful, but knowledge of the palliative care booklet was limited.

**Conclusion:**

Palliative care remains necessary for HIV despite the increasing availability of ART. However, it is currently significantly lacking in Lesotho and many other sub-Saharan African countries. Greater understanding of palliative care amongst health workers is required, as well as strong political will from the Ministry of Health. The IMAI guidelines are a useful tool for holistic HIV care, including palliative care, but they need to be used more effectively. As ART is becoming increasingly available worldwide, the complex chronic care issues for patients with HIV/AIDS should not be neglected.

## Background

Lesotho is a country of 1.8 million people, and has the third highest HIV prevalence in the world, at 23% of adults [1]. Only 26% of adults and children in need of ART were obtaining it at the end of 2007 [2], contributing to the approximately 18,000 AIDS-related deaths annually [3]. Therefore, there is clearly a continuing need for palliative care towards the end of life.

WHO definition of palliative care:

**Palliative care is an approach which improves the quality of life of patients and their families facing life-threatening illness, through the prevention, assessment and treatment of pain and other physical, psychosocial and spiritual problems **[4].

In addition to end-of-life care it is recommended for several reasons that, despite the introduction of ART, palliative care is beneficial throughout the disease trajectory.

Firstly, palliative care is necessary in order to provide social, emotional and spiritual support for both the patient and family, from the time of diagnosis [5].

Secondly, many patients present late, when they already have advanced disease and may be suffering from painful opportunistic infections [6].

Thirdly, 10% of HIV-positive patients may develop immune reconstitution inflammatory syndrome (IRIS). IRIS is the clinical deterioration resulting from sub-clinical or previously treated opportunistic infections after initiation of ART [7]. Many other patients experience undesired side effects and drug toxicities [8].

Fourthly, HIV-related cancers, such as Kaposi's Sarcoma, persist in spite of ART, and these are often not treatable in resources-poor areas [8]. Moreover, some other HIV co-morbidities may actually have increased in prevalence with increasing ART availability. This is likely to be attributable to increasing survival rates [8] and disease manifestations which occur at higher CD4 counts, such as tuberculosis and advanced cervical carcinoma [9].

Finally, although patients are likely to be pain-free once established on and remaining adherent to ART, the absence of 3^rd ^line treatment options in many areas is likely to result in the need for palliative care in cases of treatment failure [10].

In Lesotho, palliative care has only recently been introduced. There is thus no coordinating organisation and the Ministry of Health has not yet appointed a full-time palliative care lead.

The WHO recommends this integrated approach to HIV/AIDS care and has produced guidelines to facilitate its development in resource-poor settings. The IMAI guidelines are broad and holistic, emphasising decentralisation of ART provision, community care and capacity building. The acute care guidebook covers most common illnesses, including HIV-related opportunistic infections. The on-going care of HIV is covered in the "chronic HIV care with ARV therapy", and "palliative care: end-of-life care and symptom management" guidebooks. The palliative care guidebook is recommended for acute and chronic illness, and includes an illustrated guide to the WHO pain ladder and morphine administration. In addition there is a booklet for caregivers. IMAI has been, or is being, adapted and used in training in many low income countries [11]. It was adapted for the Lesotho context and implemented in 2005 [12].

Although extensive literature documents the benefits of palliative care throughout the disease trajectory, very little of this research has been undertaken in sub-Saharan Africa, and the opinions of health workers towards this type of care, and the use of IMAI, has not really been considered.

This study therefore, aimed to:

• Understand the perceptions of nurses of palliative care in Lesotho, in the era of ART availability. (Although many HIV-positive people in Lesotho are not able to access ART, the nurses involved in this study were working in hospitals where ART is available for all patients.)

• Discuss the need for palliative care, through analysis of the components of the WHO definition.

• Explore whether the nurses think that the WHO's IMAI Guidelines are a useful tool for the implementation and scaling-up of palliative care services in Lesotho.

## Methods

### Study design

This qualitative study used semi-structured interviews with 10 nurses and 6 key informants for data collection.

### Setting

The study took place in two hospitals in Maseru district in the west of Lesotho. St Josephs Hospital in Roma and Scott Hospital in Morija are both run by the Christian Health Association of Lesotho (CHAL), in conjunction with the Ministry of Health (MoH).

### Sample

The MoH and CHAL key informants were interviewed to gain an understanding of the national situation with regards to palliative care, and to enable a comparison with the ground-level hospital work occurring. The nurse participants were chosen because they had either been trained in palliative care or IMAI. Participants were selected on the advice of the CHAL executive director, as well as the medical superintendents at both hospitals.

### Data collection

Semi-structured interviews were conducted using a flexible interview guide, based on a previous literature review. Each of the interviews lasted approximately 30 minutes. The interviews were recorded digitally and subsequently transcribed verbatim.

### Analysis

Thematic content analysis was performed on the data. Themes were identified, which were based on both *a priori *issues as well as emerging ideas from the data. These were continually refined throughout the research process. Textual coding and subsequent charting was used to segregate the text into sets of data corresponding to the themes. Finally, conclusions were drawn from the patterns and consistencies that were found within the data.

### Research ethics

Participation in this study was entirely voluntary, and no incentives were given. All personal information about the participants has remained confidential at all times. Informed consent was obtained at the time of the interview. Interview recordings and transcriptions are anonymously labelled. The study gained ethical approval from the University of Leeds Ethics Committee and the Research and Ethics Committee of the Lesotho Ministry of Health.

## Results

Three primary themes were utilised throughout the study in order to consider all aspects of the WHO palliative care definition, as well as other issues which became important in the interviews:

◦ Palliative care: end-of-life care or chronic care?

◦ The need for palliative care

◦ IMAI: a tool for palliative care provision?

### Palliative care: end-of-life care or chronic care?

According to the key informants, introduction of palliative care to Lesotho began in 2006, when a situation analysis was undertaken. Consequently, it was discovered that knowledge of palliative care amongst health workers was low. In 2008, the first specific palliative care workshop was held in Lesotho, to which 18 nurses attended. This workshop was conducted by a visiting palliative care advisor from Swaziland.

For this study, it was decided that nurses should be interviewed as they constituted the largest group of health professionals receiving the palliative care and IMAI training.

In order to understand perceptions of palliative care in Lesotho, the nurse participants were initially asked what they understood by the term palliative care. This was done to assess whether views were currently oriented towards traditional end-of-life care, or towards more integrated chronic care. It was also an attempt to provide a base of understanding for the rest of the interview.

There was wide variation in the level of understanding of palliative care amongst the nurses. Several were unsure:

"It's the care given to the patient at home"

"I think it's the care that is given for HIV-positive patients, going together with TB"

"With the palliative care, I don't know anything"

Lots of the nurses described palliative care as chronic care:

"I think it's the...dealing with the...patients...with chronic illness, from the time I met until the end".

"In palliative care, that is where we give care to...chronically ill patients...and especially whereby they, the treatment cannot cure them. It's just to make them comfortable."

When asking the nurses why they thought palliative care was necessary for HIV-positive patients, end-of-life care was also recognised as an important time of need:

"I would say, since it's a chronic condition, there are still some patients who come rather late for HIV treatment, and definitely some of those, when they come late, they will still need to be cared for, in the palliative care programme."

"I think it's necessary, especially when they are in the AIDS stage...when

they, they are...lying there...when we are doing everything for them."

Knowledge of the term palliative care was clearly dependent on the level of training the nurse had received. The following definition was given by one of the two nurses who had been to this training, which is obviously very similar to the WHO definition:

"Palliative care, it is the total care which improves quality of life for those with the life limiting illness. It is primarily aimed at relieving suffering from total pain, which could be physical, emotional, social or spiritual"

Among the nurses who understood palliative care, it was clear that many thought the integrated approach could significantly improve overall care of the patients. They felt that palliative care must be holistic and that is has the potential to improve adherence to ART as well as allowing a support system for both the patient and the family, throughout the disease trajectory:

"Yes, especially, if the palliative care...was...started from the diagnosis then I think even the adherence to treatment would improve."

"Actually, the disease is lifetime... I think it would improve the psychological preparedness of the patient...it helps one to plan the way of life...and the family too...to be aware of what is in store for them and the patient"

The nurses were asked whether they believed that palliative care was still necessary, even though most of their patients were able to access ART. Many nurses felt that there remained a need for palliative care, as it could be beneficial for pain and symptom management. It was also thought to provide an opportunity to give psychosocial support to the patient and family:

"Yes, because they are still having these symptoms, like painful feet, so we have to tell them that no, you have to expect death, the symptoms, the side effects, and even the drugs, if the pain is not subsiding, we have to increase the dose."

"Because starting from the beginning when you first meet the patient...if I can start off by doing the testing, the patient will react...they react differently...and they need...counselling...on and on...until they get used to the idea of being positive. Then, when you prepare her for the drugs... even that taking of ART is still a problem...they need more help, more support, from all of us...even the relatives should be part of it. So, when they are even chronically ill, the chronic illness takes all, all the person's life. The person should be helped, really...to go on with that."

### The need for palliative care

The need for holistic palliative care for HIV-positive patients, which includes pain, symptom and psychosocial management, has already been discussed. In the interviews, many HIV-related symptoms were described, and the need for psychosocial support for both the patient and their family was recognised. However, during most of the interviews the issue of pain management was highlighted repeatedly; this section therefore, will mostly focus on that topic.

Discussions were initially held with staff at the Ministry of Health. From these interviews it was evident that the pain assessment skills of health professionals are inadequate, resulting in the under-prescription of strong analgesia. Morphine is available in the country but only in tablet form, and these can only be prescribed in hospitals. Current government plans to improve the situation include changes to the Drugs of Abuse Act, and the introduction of morphine at the health centre level. The African Palliative Care Association has suggested to the Ministry of Health that they should be aiming to increase their country's quota of morphine. Currently the national consumption is very low, and the country's total stock is not consumed annually.

All of the nurses interviewed agreed that some HIV-positive patients suffer from pain. However, there was variation in the significance of the pain described, as well as the prevalence of pain in patients who are taking ART. Some nurses stated that patients do not experience pain once treatment is initiated. Others suggested the opposite and additionally thought that the pain may often result from the treatment itself:

"The pain is from the knees and the feet... the neuropathy, peripheral neuropathy, that's the main cause of the pain."

"Yes...many complain of pain...general body pains...but, there are some who would say they don't feel any pain, especially when they are already on [antiretrovirals]. They will say the pains have gone, they no longer need the painkillers."

"Usually, those patients who are on a regimen that includes D4T, that is the one that is causing the pain. It is the neuropathic pain"

It was acknowledged by some nurses that pain is not treated well in Lesotho. This was attributed to lack of appropriate analgesia available, and inadequate knowledge of pain management:

"Not really... we don't have the strong enough analgesia...but you have to counsel them before...to tell them that, because, patients with HIV and AIDS, patients are normally complaining of painful feet...but...though people give us some...some of the pain goes easily...but some doesn't."

"No...In Lesotho, we expect the patients to respond...to the first step of the ladder [the WHO pain ladder] ...but actually, people experience pain differently...in different ways. Some actually need stronger [painkillers]."

Contradictorily, other nurses thought that the analgesia available was adequate, and stronger pain relief was not needed for their patients:

"We have brufen, we have panado, and we have panado with codeine in it... [It treats the pain] very well, really, especially the brufen. They will say it makes them feel better."

Several nurses thought there was a need for morphine for HIV-positive patients, whilst acknowledging that the number in need was not large; others recognised a need for morphine in the hospital, but not necessarily for patients with HIV.

"I don't think there are that many, but there are patients, who really are very critical, and are having too much pain and so forth."

"Yes, I think we should have it...though most of them, the unbearable pain they get is...it is usually the headache. It is usually related to meningitis, though I'm not sure whether someone with meningitis can be treated with the morphine, but I just think it should be in the hospital.

It was suggested by several nurses that morphine was mainly needed by patients with cancer, including those with HIV-related cancer:

"Yes, something like the cancer people, those who are suffering from lung cancer, and liver cancer...and so we don't have morphine, we need it..."

"Oh, another thing, with these HIV-positive patients, sometimes you find that...the cancer, comes in also... so these people, when they have cancer, sometimes they feel the severe pain. So then they might be needing the morphine."

In Lesotho all hospitals are supposed to have morphine available, however, none of the nurses thought it was available where they were working:

"What we were taught is that we should give opiates, opioid and opium...morphine... but those drugs we don't have. Yes. We only have painkillers. Some mild painkillers, like paracetamol, epi-codeine, and the panado...yes we don't have that stuff that we talked about, here."

Suggested reasons for the unavailability of morphine included a fear of addiction, cost and under-prescription:

"We, we feel...fear. That people will be addicted...that people will do the...the fear of addiction...the fear of drugs falling in inappropriate hands...yes...there are so many fears... Because it also impacts on prescription of these drugs. The physicians can be reluctant to prescribe the morphine."

"I think it's because it's expensive, though I don't know how much it even costs."

"Under-prescribing... It can be available for...if it's prescribed. Because, the availability depends on how much is prescribed."

Other symptoms were described including nausea, diarrhoea and rashes. In contrast to pain however, these were thought to be well treated and the nurses felt they had enough resources available to be able to do so:

"If they have diarrhoea, we treat the diarrhoea, if they are vomiting we treat the vomiting, if the rash, we give something for the rash."

Finally, it was emphasised by several nurses that there was a need to recognise the non-physical pain associated with HIV, as well as providing psychosocial support for both the patients and families. For example, one nurse stated the following:

"The...it's pain...from when a patient gets diagnosed...it could not be physical pain...but the pain that comes from getting a... that comes from being diagnosed with a...life-threatening illness...I think, the pain, it should be addressed more holistically."

### IMAI: a tool for palliative care provision?

IMAI was described in the key informant interviews as being at an early stage in Lesotho. It was mentioned that although the training had been undertaken already, palliative care was only briefly discussed, and hence understanding of this by nurses remains low. It was suggested that improvements needed to be made to the IMAI training course, to ensure greater understanding of palliative care. It was also noted that although these training sessions can be useful, some nurses may be overwhelmed by the large number of new protocols introduced in Lesotho. One informant commented that the title of the guidebook "Palliative care: end-of-life care and symptom management" is "scary", and it was suggested that this was perhaps why it failed to be used. This participant also suggested that if nurses are treating patients with ART, they will ignore this guidebook because they believe that palliative care is not necessary unless the patient is dying, resulting in the neglect of the important sections on pain and symptom management.

It appeared that several of the nurses had attended either the IMAI training, or the palliative care workshop. It seems that it would be more useful to combine these sessions, to ensure that the integrated approach to care is fully understood.

Attempts were made to only interview nurses who have been trained in IMAI; therefore knowledge of the existence of IMAI was high among the participants. Training was described as mostly focussing on ART provision. Most of the nurses thought that using the guidelines improved clinical care for the patients and found them to be very useful. However, it was not clear how many of the nurses regularly used the guidelines in practice. Most nurses said it was used, but very few of them appeared to have a copy available. Only one nurse admitted that perhaps they are not always used:

"We don't really read them. Not all the time. They are useful, really...but it's just that, we never read them from A to Z so that...you read them only when you meet a problem, so that you say, where are the guidelines?"

When asked whether the training included the palliative care module, most nurses either hadn't heard of it, or couldn't remember what it had involved. Only one nurse thought she had a copy of the guidebook, but this was always left at home.

"They didn't. It only appeared, like I said, when we were talking about the issue of initiating the ARV's, and initiating the treatment supporter... relating well with the patient, so that the counselling, it's on-going, such things... but not a specific topic on palliative care"

"No, we were given it [the palliative care module guidebook], but I'm not sure, we didn't receive the training."

It was often mentioned that a specific workshop had been held for palliative care and thus it was not included in the IMAI training. But a senior nurse in one hospital explained the following:

"The training was given...it is a module of the IMAI guidelines. But the nurses sometimes they have the knowledge...but they still don't always understand the palliative care, they do not take it holistically."

Finally, when asked whether more palliative care training would be useful, the following nurse made some very important points:

"I think so...yes...I think we should...like...HIV it's for everybody. We have this Know Your Status Campaign, whereby the people in the village test the patients, the people in the village... so if palliative care is there, maybe they would be supported to grow, not only to do VCT and refer to the hospital, to have some things that they can do after testing...the follow-up after they have sent their patient to the clinic, and then when they come back they know what to do...unlike now when they just test, and say go to the hospital. Village health workers are the only people I can think of that can make it happen, because they are long in the field, and they know the villagers well...they get in contact with the people that know....like the doctors and the nurses...and so they can refer the patients if they need to."

## Discussion

It should be appreciated that chronic care is a relatively new field of health care in most of sub-Saharan Africa. Most facilities have concentrated on acute care, leaving several specialist centres, for example Mildmay in Uganda, to provide chronic and palliative care [13]. From the results it may appear that some of the nurses had very little knowledge of palliative care, however this is perhaps related to a difference in terminology. If the definition of palliative care is broken down, and all components are examined, it is apparent that this type of care may exist in Lesotho, and that nurses may be performing some aspects of it routinely, but without thinking of it as 'palliative care'. Palliative care was originally developed in the UK [14], so it is essentially a Western construct. Therefore, during the interviews all aspects of the palliative care definition were explored, rather than assuming knowledge of and relying on the exact term 'palliative care'. Nevertheless, one senior nurse suggested that although this may be the case, an increased understanding of the concept of palliative care could be beneficial in improving the holistic care of the patient. In addition, many of the nurses did not feel they had enough staff or time to be able to provide this type of necessary care.

Discussion of the results will be divided into the three themes outlined previously.

### Palliative care: end-of-life care or chronic care?

It is increasingly recognised that the traditional distinction between early-stage and late-stage HIV-disease, with palliative care reserved for end-of-life care, is invalid. Pain and symptom control, in addition to psychosocial support, are essential throughout the disease.

Interestingly, despite these relatively new recommendations, the nurses in Lesotho associated palliative care more with this integrated approach than with the traditional approach. Many felt the benefits of integrated care could improve the overall quality of life of the patient and family.

It has been suggested that since the introduction of ART, AIDS care has been, unintentionally, detached from palliative care. Palliative care should be a holistic practice, and ART is essentially just one aspect of this. HIV has increasingly been viewed as a chronic and manageable condition, but it remains incurable. It has been recognised that AIDS care has become increasingly biomedical and perhaps the overall aim of good chronic care, which should be sought for patients with an incurable condition, is being neglected [15].

It seems that in Lesotho, the nurses contradict this, in their opinions at least. Most recognised the necessity of palliative care, even for patients taking ART. However, it is unclear whether this type of comprehensive chronic care is actually occurring in practice, and whether it is even possible considering the severe staff shortages.

### The need for palliative care

Pain is reportedly one of the most prevalent symptoms in patients with HIV, throughout the course of the disease, and especially in the later stages [16]. Several reports note that between 30% and 90% of patients with AIDS suffer from pain at some point in their illness [17-20]. Additionally, it is suggested that 1.84 million people in sub-Saharan Africa died from AIDS in pain in 2003 [21]. Even where ART is available, pain remains a problem. For example, patients can experience a severe headache from cryptococcal meningitis during immune reconstitution after commencing ART. Additionally, the ART stavudine (d4T) can result in neuropathic pain.

Pain was described by nurses as an issue for HIV-positive patients in Lesotho. However, the responses varied in terms of the prevalence and severity of the pain. This could simply be indicative of the varied nature of patients' symptoms in practice.

Pain control is an essential component of palliative care provision, as it is very difficult to manage psychological and other problems if the patient is in pain. Research in sub-Saharan Africa has found that pain in patients with HIV is often poorly managed and under-treated [22].

Although morphine is recommended for patients with HIV by the WHO [23], several of the nurses in this study thought that it was not needed. However, this may be invalid evidence, as pain assessment has been described as poor in Lesotho. It is therefore difficult to ascertain what the actual pain management needs are in Lesotho.

Discussion revealed that morphine is not readily available in hospitals in Lesotho, despite its theoretical availability. Reasons for this were mostly centred on under-prescription by physicians. This in turn was related to a reluctance to prescribe opiates, attributed to fear of addiction and dependence. Lesotho is not unusual. It is well documented that poor access to morphine has partially resulted from fears of addiction amongst health professionals and policy makers [24]. However, a research study of over 10,000 patients reported that iatrogenic opiate addiction rates were less than 1% [25]. Cost was described as another obstacle to morphine prescription. However, there is no evidence for this; at only 1 US cent for 10 mg of generic morphine sulphate tablets [26] they are very affordable and hence cost should not constitute a reasonable barrier.

Appropriate psychosocial care remains inadequate, and is often limited to pre- and post-test counselling only. Health staff shortages significantly contribute to this problem, thus greater responsibility should be given to community health workers to perform this role.

HIV is often prioritised in Lesotho; therefore most of the discussions surrounding palliative care were focussed on HIV. However, there are many other significant diseases and conditions, particularly cancers, for which there is an equal, if not greater, need for palliative care.

### IMAI: a tool for palliative care provision?

Guidelines have been shown to be effective in clinical care, as long as they are credible and acceptable to those using them [27]. However, it has also been found that guidelines should not be introduced without supervision and audit [28]. It has been suggested that disseminating the information and training the staff alone, is not sufficient for ensuring better performance.

This research study has shown that the IMAI guidelines are acceptable and credible to the nurses interviewed. However, there appeared no signs of further monitoring or on-going training after its initial introduction, arguably increasing the likelihood of nurses not regularly using these guidelines in their practice. Nevertheless, it is important to note that the training of nurses in ART provision, through IMAI, has been effective in increasing knowledge of treatment regimes and associated problems.

Through observation whilst in Lesotho, it was evident that there are a significant number of training workshops for nurses, and these often last for one or two weeks. The usefulness of these workshops, and the strain of staff being away from work needs to be assessed, and a suitable solution explored to enable effective and efficient staff training, whilst maintaining the care of the patients during the training periods.

It was repeatedly explained that the IMAI training had mostly focussed on ART provision and therefore the knowledge of the palliative care module was very low. This supports the assertions made previously; that AIDS care seems to be very treatment-focused with less emphasis placed on chronic and palliative care.

### Limitations

The nurses interviewed were working in hospitals where patients have access to ART, thus this is not necessarily a true representation of the situation throughout Lesotho. The study does not take into account the perspectives of nurses working in more rural settings and where ART is not available.

The participants used in this study were suggested by CHAL and the medical superintendents of the hospitals, which may have resulted in selection bias. However, there were only a limited number of nurses who could have taken part, due to the requirement that they had attended one of the training sessions.

### Recommendations

It is essential that knowledge of pain management amongst health workers is improved. This needs to be introduced into undergraduate nurse training, as well as refresher courses after qualification. However, this post-graduate training should occur in the health facility setting, to avoid interruption of clinical services and exacerbation of staff shortage problems [29]. Doctors also need to receive further training on palliative care. This would only be possible in the post-graduate situation, as there is no medical school in Lesotho. Training should be followed up with outcomes assessment, within a specified period.

Palliative care training should not be viewed as separate from IMAI. However, it will also be important to ensure that future palliative care training sessions include some clinical aspects with patient involvement. Only through demonstrating in a clinical setting how palliative care can be effectively performed will the level of its provision improve.

Morphine should be available in all hospitals and health centres and in the community; whilst ensuring that there is close collaboration between these health service levels. Plans are currently in progress for introducing morphine in health centres in Lesotho, which should increase availability in the more rural areas. However, there needs to be a change in the perception of physicians towards morphine, to increase the amount that is prescribed. Indeed, morphine could also be made more available, in countries such as Lesotho, by allowing nurses to prescribe it. This has been introduced in Uganda, and has been relatively successful [30].

Finally, greater emphasis must also be placed on increasing the availability of effective psychosocial care, as part of palliative care. This should be both health facility and community-based. Community-based care, including work by community health workers, will also help to take some of the strain of the overburdened health system.

To build upon this study, a larger, more representative sample could be used, using both qualitative and quantitative methods of data collection. Further research areas include: actual pain management needs; palliative care needs in regions without ART availability; quantifiable benefits of the integrated approach to palliative care compared to traditional end-of-life care.

## Conclusion

Palliative care remains necessary in the era of ART availability, for improving the quality of life of the patients and their families. But, it is significantly lacking; both in Lesotho and in many other sub-Saharan African countries. This is likely to remain the situation until there is strong political will from leaders in the Ministry of Health.

The approach taken should be integrated and holistic. In addition, pain management must be improved through increasing knowledge of pain assessment and treatment, and by making morphine more accessible and available.

IMAI (acute, chronic HIV-ART and palliative care guidebooks) is a very useful tool for scaling up HIV care, including palliative care, provision. But, in our opinion, it is not being adequately used. There needs to be a greater focus on providing a comprehensive care package, to avoid the neglect of palliative care, and pain and symptom management.

With the international drive to provide universal access to ART worldwide, the important holistic approach to care for patients living with HIV/AIDS must not be forgotten.

## Competing interests

The authors declare that they have no competing interests.

## Authors' contributions

MK helped design the study, perform the interviews and data analysis and write the draft manuscript. JW contributed to the conception and design of the study, facilitated the research and edited and refined the manuscript. All authors read and approved the final manuscript.

## Pre-publication history

The pre-publication history for this paper can be accessed here:


